# Successful treatment of a benign, non-infected cyst in a dog by bone marrow injections

**DOI:** 10.17221/19/2023-VETMED

**Published:** 2023-08-21

**Authors:** Beata Degorska, Jacek Sterna, Joanna Bonecka, Malgorzata Sobczak-Filipiak, Jowita Jacewicz

**Affiliations:** ^1^Department of Small Animal Diseases and Clinic, Institute of Veterinary Medicine, Warsaw University of Life Sciences – SGGW, Warsaw, Poland; ^2^Department of Pathology and Veterinary Diagnostics, Institute of Veterinary Medicine, Warsaw University of Life Sciences – SGGW, Warsaw, Poland

**Keywords:** bone defect, bone regeneration, canine, humerus, mini-invasive treatment

## Abstract

Bone cysts are rare orthopaedic problems in dogs. There are no clear treatment guidelines. A young male Shih Tzu was referred to Small Animal Clinic with fifth-degree lameness (5/5) of the left thoracic limb, and with swelling and deformation of the distal humeral region. The radiological assessment revealed an enlargement of the distal brachium and an extensive hypodense osteolytic lesion in the distal metaphyseal region of the humerus. Diagnosis of the bone cyst was formulated and treated with a mini-invasive method using autologous non-concentrated bone marrow injections. The treatment was successful, and at the three weeks, the cyst significantly changed its structure. The follow-up at 12 weeks after the first injection, and at one year revealed complete recovery. To our knowledge, this is the first evidence of a bone cyst in a young dog successfully treated with a minimally-invasive method by using a non-concentrated autologous bone marrow injection.

Bone cysts are intraosseous, tumour-like, chambered cavernous spaces containing serosanguineous fluid, with fibrous connective tissue on the internal surface of the cyst (Petazzoni et al. 2015).

The prevalence of bone cysts is unknown. Three main groups of bone cysts are distinguished: simple cyst [solitary bone cyst (SBC), previously known as a unicameral bone cyst (UBC)], aneurysmal bone cyst (ABC), and others (e.g., in the course of osteoarthritis, post-traumatic, intraosseous lipoma, mucous cyst, epidermal cyst) (Doganavsargil et al. 2015; Vagias et al. 2020). In human medicine solitary bone cysts are benign, and 70% of them are diagnosed in children, more than 95% arise from the metaphysis, and they occur in the proximal humerus or femur. There are multiple hypotheses about solitary bone cyst formation. The current literature describes this solitary bone formation as a blockage or occlusion of the intramedullary circulation (Rosenblatt and Koder 2019). Aneurysmal bone cysts are expansile, osteolytic lesions, containing thin, ballooned periosteum (“soap-bubble appearance”), with large vascular sinusoids, separated by septa. Dynamic lifting of the periosteum produces new bone growth on the periphery of the lesion. Pathological fractures caused by localised bone destruction are common. Histopathological examination shows fibrous and osseous septa lined by fibroblasts and multinucleate giant cells with blood-filled cystic areas. Local recurrence of ABCs can be observed both in human and veterinary medicine (Duval et al. 1995; Barnhard 2002; Dowdle et al. 2003; Salierler et al. 2004; Mankin et al. 2005; Vignoli et al. 2015; Barbanti-Brodano et al. 2017). Bone cysts are rare in dogs, however, single, solitary one bone cysts resulting in deformation of the bone shape and radiolucency in the radiographic assessment were reported in the proximal or distal metaphyseal area of long bones in young dogs (5–15 months), more commonly in males than females. Most of the solitary bone cysts are benign. They can be monostotic or polyostotic, lytic and expansile, with narrowing of the cortex, and little to no periosteal new bone formation (Thompson and Pool 2002; Sarierler et al. 2004; Petazzoni et al. 2015). Pathological fractures are often seen in these lesions if left untreated. German Shepherds, Doberman Pinchers, Mastiffs, Golden Retrievers, Shih Tzus, Siberian Huskies, and Yorkshire Terriers are most commonly affected (Halliwell 1993; Petazzoni et al. 2015; Garcia-Gonzalez et al. 2021). Solitary bone cysts have been reported in dogs’ tibia, pelvis, humerus, metatarsal and lumbar regions (Sarierler et al. 2004).

The knowledge about the aetiology and treatment of bone cysts is limited (Kadhim et al. 2014; Biezynski et al. 2015; Mascard et al. 2015; Rosenblatt and Koder 2019). The pathogenesis of bone cysts is uncertain, and likely multifactorial. However, hereditary factors altered vascularity, or trauma are probably the main cause of the condition (Garcia-Gonzales et al. 2021). Vascularity alteration leads to local circulatory disturbance, increased venous pressure, arteriovenous shunting, and the formation of blood-filled cysts in the medullary cavity. Additionally, in human medicine, chromosomal analyses indicated hereditary factors (Barbanti-Brodano et al. 2017; Rosenblatt and Koder 2019). Diagnosis can be made based on the medical history, clinical examination, clinical signs, diagnostic images [radiographic examination, computed tomography (CT) or magnetic resonance (MRI)] needle biopsy, and histopathology. Solitary bone cysts usually have typical radiographic features. Some of the cysts are unicameral, and radiolucent with a thin cortical rim and sometimes pathognomonic “fallen leaf” signs. The cavity is lined with a thin fibrous membrane, which may contain immature calcified, flakey cement-like bone matter (Doganavsargil et al. 2015; Mascard et al. 2015). Different methods have been proposed for the treatment of bone cysts, both in veterinary and human medicine. The treatment may vary depending on the aetiology, clinical signs, type of cyst, localisation, and size. Conservative treatment can be applied to small, solitary bone cysts. Bone cysts can be also treated surgically with curettage and bone grafting, sclerotherapy, cryotherapy, injection of corticosteroids, alcohol, injection of concentrated bone marrow, drilling and drainage, curettage with the introduction of autogenous or allogenous bone graft, bioapatites or bone cement, ostectomy, selective arterial embolisation, synthetic bone graft substitutes, and radionuclide ablation (Duval et al. 1995; Sarierler et al. 2004; Cho et al. 2007; Knight and Hankenson 2013; Kadhim et al. 2014; Biezynski et al. 2015; Barbanti-Brodano et al. 2017; Chahla et al. 2017; Oryan et al. 2017; Rosenblatt and Koder 2019; Garcia-Gonzales et al. 2021). The purpose of this study is to present short and long-term follow-ups of a benign, non-infected bone cyst treated with non-concentrated bone marrow injections. To our knowledge no case of bone cyst treated with non-concentrated bone marrow injection has been reported yet in veterinary medicine.

## Case description

**Figure 1:**
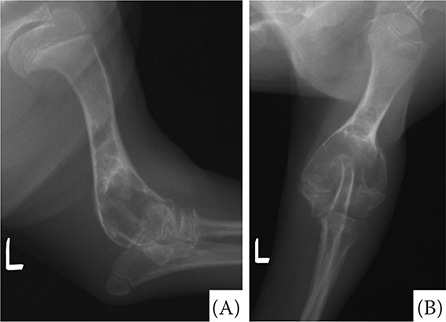
Lateral and craniocaudal radiographic view of the left humerus revealed lesions in the metaphyseal region of the distal humerus with enlargement bone outline and thinning cortical bone

Five-month-old, very active, male Shih Tzu dog, weighing 4.4 kg, was referred to Small Animal Clinic with non-weight bearing [fifth-degree lameness (5/5)] of the left thoracic limb, which appeared two weeks before the clinical examination of the dog. The lameness appeared suddenly, with no history of trauma, and gradually progressed. Swelling and deformation were noted in the distal humeral region of the left thoracic limb. Clinical evaluation of the dog showed no other abnormalities, and results from haematological and serum chemistry analyses were within the normal reference range. A radiographical assessment of the left thoracic limb revealed an enlargement of the distal humerus outline, and an extensive hypodense osteolytic lesion containing irregular radiolucency inside the distal metaphyseal region of the humerus. The lesion caused cortical thinning from 1/3 of the length of the steam to the distal physis of the humerus (Figure 1).

**Figure 2:**
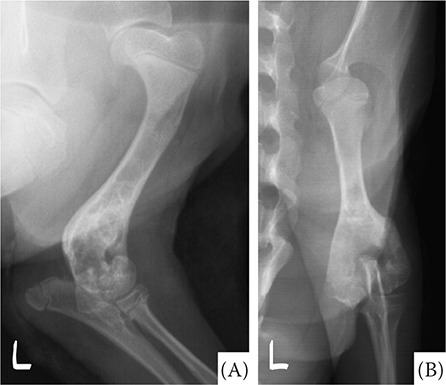
Lateral and craniocaudal radiographic view three weeks later A significantly increased bone density at the bone cyst site without periosteal reaction is visible on the lateral and craniocaudal view of the left humerus

As the lytic changes in the humerus might suggest malignancy, a chest radiographic study was additionally performed. The thoracic cavity in three views was free of metastases. The radiolucent lesions, with a narrow zone of transition, were centrally located, expanded the bone with thinning of the cortical bone, and showed a sclerotic margin with no periosteal reaction or soft tissue component. Due to the owner’s refusal to collect a specimen for histopathology (core biopsy), only samples for cytopathologic evaluation were collected. The collected fluid was serosanguineous in appearance. Cytopathological microscopic examination showed numerous red blood cells, some spindle-shaped fibroblasts, and some macrophages. There were no cells suggesting neoplasm (osteoblast, atypical mesenchymal cells) nor inflammatory cells like neutrophils, lymphocytes, or plasma cells. Neither bacteria nor infectious factors were found. Moreover, samples were sent for microbiological examinations, and bacterial and fungal cultures did not yield any growth. Based on the history, animal age, localisation of the lesion, X-ray results, and cytological findings the diagnosis of a benign bone cyst was made. Treatment by using autological non-concentrated bone marrow injections was recommended to the owner. The skin of the lateral area of the greater trochanter and the skin of the lateral area of the distal brachium were clipped. The dog was premedicated with intramuscular administration of methadone hydrochloride (0.3 mg/kg), dexmedetomidine (5 μg/kg), and midazolam (0.3 mg/kg), and induced intravenously with propofol (3 mg/kg). The skin of the clipped areas was aseptically prepared. Bone marrow sample was aspirated from the left femoral medullary canal using under trochanteric fossa approach with a dry, sterile syringe and an 18 G needle. Immediately after its collection, bone marrow was administered to the cyst by a lateral transdermal injection with an 18 G needle. The cyst wall was soft and thin. The total amount of bone marrow deposition was 2 cc. The limb was protected against possible humeral fracture using a soft cast for three weeks. The purpose of this was to immobilize the affected brachium, the bandage passed around the chest and behind the front legs, covering the whole affected shoulder and half of the left antebrachium. The restricted activity was recommended with short leash walks. Three weeks later, radiographic control was performed under sedation and significant improvement was visible with increased opacity in the cyst (Figure 2). The procedure with bone marrow injection was repeated, with the same restrictions of the activity.

**Figure 3:**
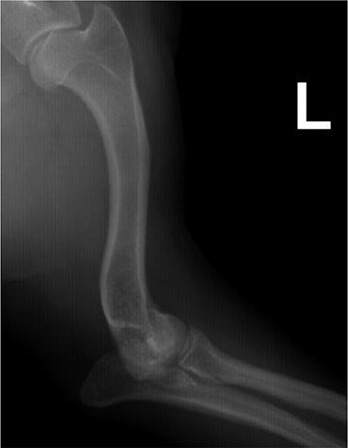
Follow-up radiographs at 12 weeks after the first bone marrow injection Note correct remodelling of the humerus and cyst healing, and the rapid healing without extensive callus

**Figure 4:**
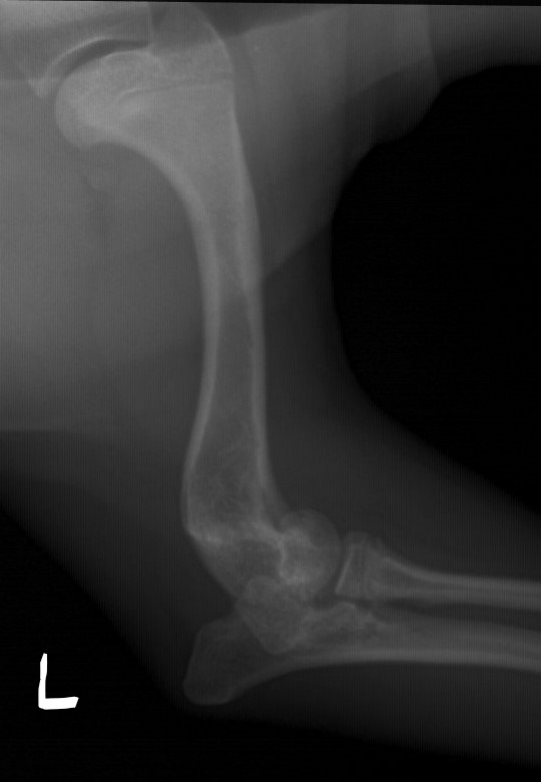
The control radiographic evaluation one year after the first bone marrow injection The radiograph shows no abnormalities with normal distal diaphysis of the humerus and normal humeral condyle with superimposed epicondyles

At three weeks following the second procedure, the dog was in very good condition and clinically had 1/5 lameness. There were no signs of acute pain or swelling. A radiographic assessment showed that the cyst lesion was no longer visible, although the distal humerus was still enlarged. At our request, the clinical and radiological examination was repeated 12 weeks after the first injection (Figure 3), and one year after the last procedure (Figure 4) to assess how the bone remodelling proceeded, and whether there were any signs of the disease.

Figure 3 shows that bone remodelling and healing were very fast. The radiographic assessment performed one year after the first injection (Figure 4) shows the humerus without any changes in the bone structure. The distal metaphysis gradually realigned and residual angulation was slowly corrected. Normal endosteal and subperiosteal surfaces of the cortex were well-defined and continuous. The remainder of the cortex was radio-opaque and homogeneous in density, the bone was normal. The dog returned to a normal activity level. No local or systemic reaction related to the bone marrow injections was detected.

## DISCUSSION

The aetiology of bone cysts is unclear. They may occur after injury, due to fibrous dystrophy, abnormal bleeding, blood pressure increased, deficient blood supply, ischemic cartilage necrosis, or bone tumour (Barnhart 2002; Dowdle et al. 2003; Olstad et al. 2015; Garcia-Gonzales et al. 2021). The cortical wall thinning and radiolucent lesion are typically visible on the radiographic examination. In the presented case, the diagnosis was made based on the history, clinical examination, radiographic diagnostic imaging, fine needle aspirate biopsy, and microbiological samples collected from the cyst. Fine needle aspirates are unlikely to be useful in the diagnosis of such bone changes (Craig et al. 2016). Usually, they are heavily contaminated with blood, so confirmation requires histopathological diagnosis of biopsy specimens. In this case, the owner of the dog disagreed with the core biopsy; so the fine needle aspiration biopsy was the only possible way to verify the clinical diagnosis. There were no cells, suggesting neoplasm (osteoblasts, atypical mesenchymal cells) nor inflammatory cells, like neutrophils, lymphocytes, plasma cells, bacteria, and other infectious factors. CT or MRI allows a better description of the three-dimensional architecture of the cyst and the humeral bone, but neither CT nor MRI is considered essential to establish the size and localisation of the cyst. One of the treatment options for the solitary bone cyst is concentrated bone marrow injections which were reported to yield promising results in human medicine. This method of treatment requires laboratory efforts to obtain a bone marrow concentrate ready for administration (Cho et al. 2007; Barbanti-Brodano et al. 2017; Chahla et al. 2017). The injection of concentrated autologous bone marrow into the cyst may be combined with a corticosteroid agent (Cho et al. 2007). Bone marrow is an osteogenic material, but not as good as a cancellous bone graft (Kraus and Kirker-Head 2006). Administration of bone marrow taken directly from the proximal part of the long bone is a minimally invasive technique in dogs, very easy to prepare and perform, and it does not require specialised equipment. Typically, bone marrow is aspirated from the proximal humerus, proximal femur, or tuber coxae. In veterinary patients, mesenchymal stem cells are mainly used for the treatment of tendon, ligament, or cartilage-joint (including osteoarthritis) injuries in dogs and horses. There are some publications describing the use of bone marrow to facilitate fracture healing (Crovace et al. 2008; Fortier and Travis 2011). Bone marrow accounts for 2% of the dog’s body weight. It consists of haematopoietic islands, adipose cells, and vascular sinuses suspended in the trabecular bone (Travlos 2006). Bone marrow stem cell populations differentiate between haematopoietic and nonhaematopoietic. The second group includes endothelial progenitor cells (EPCs) and mesenchymal stem cells (MSCs). Mesenchymal stem cells are progenitors for chondroblasts, osteoblasts, and adipocytes. Tissue-specific CXCR4^+^ cells and cytokines that are also found in the bone marrow, play a crucial role in the regenerative and repair processes of tissues as they mobilize progenitor cells (Bianco et al. 2008; Knight and Hankenson 2013; Oryan et al. 2017). Transplanted bone marrow stem cells help activate damaged tissue regeneration. The use of bone marrow transplantation is an extremely exciting innovation in wound care research and reconstructive tissue lesions.

The aim of this study is to present a full recovery of a benign, non-infected bone cyst in a young dog, including the long-term follow-up (one year). To our knowledge, there have not been any reported cases in which fresh bone marrow has been used. Non-concentrated marrow injections were effective in the treatment of a bone cyst and led to complete recovery in a short time with dynamic healing of the bone defect. This procedure is minimally invasive, with no known side effects, and can be performed without any special equipment. It seems to be a promising alternative to the methods previously described in the literature but requires further research and observation. The limitation of our case report was the lack of histopathology. The owner refused a core biopsy to be performed, and our preliminary diagnosis was based on history, clinical examination, radiological evaluation, and cytopathology. It led us to an empirical approach to the treatment, which was successful.

## References

[R1] Barbanti-Brodano G, Girolami M, Ghermandi R, Terzi S, Gasbarrini A, Bandiera S, Boriani S. Aneurysmal bone cyst of the spine treated by concentrated bone marrow: Clinical cases and review of the literature. Eur Spine J. 2017 May;26(Suppl_1):158-66. 28168344 10.1007/s00586-017-4978-x

[R2] Barnhart MD. Malignant transformation of an aneurysmal bone cyst in a dog. Vet Surg. 2002 Nov-Dec;31(6):519-24. 12415520 10.1053/jvet.2002.36014

[R3] Bianco P, Robey PG, Simmons PJ. Mesenchymal stem cells: Revisiting history, concepts, and assays. Cell Stem Cell. 2008 Apr 10;2(4):313-9. 18397751 10.1016/j.stem.2008.03.002 PMC2613570

[R4] Biezynski J, Piatek A, Skrzypczak P, Oginska O, Kielbowicz Z. Operacyjna metoda leczenia cyst kostnych u psow przy uzyciu cementu kostnego [Operating method for treatment of bone cysts in dogs with bone cement]. Med Weter. 2015;71(6):390-2. Polish.

[R5] Chahla J, Mannava S, Cinque ME, Geeslin AG, Codina D, LaPrade RF. Bone marrow aspirate concentrate harvesting and processing technique. Arthrosc Tech. 2017 Apr 10;6(2):e441-e5. 28580265 10.1016/j.eats.2016.10.024 PMC5443590

[R6] Cho HS, Oh JH, Kim HS, Kang HG, Lee SH. Unicameral bone cysts: A comparison of injection of steroid and grafting with autologous bone marrow. J Bone Joint Surg Br. 2007 Feb;89(2):222-6. 17322439 10.1302/0301-620X.89B2.18116

[R7] Craig LE, Dittmer KE, Thompson KG. Bones and joints – Tumor-like lesions of bones. In: Maxie G, editor. Jubb, Kennedy and Palmer’s pathology of domestic animals. St. Louis, MO: Elsevier; 2016. p. 125-7.

[R8] Crovace A, Favia A, Lacitignola L, Di Comite MS, Staffieri F, Francioso E. Use of autologous bone marrow mononuclear cells and cultured bone marrow stromal cells in dogs with orthopaedic lesions. Vet Res Commun. 2008 Sep;32:S39-44. 18688750 10.1007/s11259-008-9095-1

[R9] Doganavsargil B, Ayhan E, Argin M, Pehlivanoglu B, Kececi B, Sezak M, Basdemir G, Oztop F. Cystic bone lesions: Histopathological spectrum and diagnostic challenges. Turk Patoloji Derg. 2015;31(2):95-103. 25652560 10.5146/tjpath.2014.01293

[R10] Dowdle SM, Spotswood TC, Lambrechts NE, Duncan NM. Aneurysmal bone cyst in the distal radius of a dog: Diagnostic imaging and surgical treatment. Vet Comp Orthop Traumatol. 2003;16(2):116-21.

[R11] Duval JM, Chambers JN, Newell SM. Surgical treatment of an aneurysmal bone cyst in a dog. Vet Comp Orthop Traumatol. 1995 Apr;8(4):213-7.

[R12] Fortier LA, Travis AJ. Stem cells in veterinary medicine. Stem Cell Res Ther. 2011 Feb 23;2(1):9. 21371354 10.1186/scrt50 PMC3092149

[R13] Garcia-Gonzalez M, Munoz Guzon FM, Gonzalez-Cantalapiedra A, Lopez-Pena M, de Frutos Pachon F, Pereira-Espinel Plata T, Gonzalez Fernandez PM, Serra Rodriguez JA. Case report: First evidence of a benign bone cyst in an adult Teckel dog treated with shark teeth-derived bioapatites. Front Vet Sci. 2021 Feb 22;8:1-6. 10.3389/fvets.2021.626992 PMC7937721 33693042

[R14] Halliwell WH. Tumorlike lesions of bone. In: Borjab J, editor. Disease mechanisms in small animal surgery. 2^nd^ ed. Malvern: Lea & Febiger; 1993. p. 932-43.

[R15] Kadhim M, Thacker M, Kadhim A, Holmes L Jr. Treatment of unicameral bone cyst: Systematic review and meta analysis. J Child Orthop. 2014 Mar;8(2):171-91. 24570274 10.1007/s11832-014-0566-3 PMC3965764

[R16] Knight MN, Hankenson KD. Mesenchymal stem cells in bone regeneration. Adv Wound Care (New Rochelle). 2013 Jul;2(6):306-16. 24527352 10.1089/wound.2012.0420 PMC3842877

[R17] Kraus KH, Kirker-Head C. Mesenchymal stem cells and bone regeneration. Vet Surg. 2006 Apr;35(3):232-42. 16635002 10.1111/j.1532-950X.2006.00142.x

[R18] Mankin HJ, Hornicek FJ, Ortiz-Cruz E, Villafuerte J, Gebhardt MC. Aneurysmal bone cyst: A review of 150 patients. J Clin Oncol. 2005 Sep 20;23(27):6756-62. 16170183 10.1200/JCO.2005.15.255

[R19] Mascard E, Gomez-Brouchet A, Lambot K. Bone cysts: Unicameral and aneurysmal bone cyst. Orthop Traumatol Surg Res. 2015 Feb;101(Suppl_1):S119-27. 25579825 10.1016/j.otsr.2014.06.031

[R20] Olstad K, Ostevik L, Carlson CS, Ekman S. Osteochondrosis can lead to formation of pseudocysts and true cysts in the subchondral bone of horses. Vet Pathol. 2015 Sep;52(5):862-72. 25428408 10.1177/0300985814559399

[R21] Oryan A, Kamali A, Moshiri A, Baghaban Eslaminejad M. Role of mesenchymal stem cells in bone regenerative medicine: What is the evidence? Cells Tissues Organs. 2017;204(2):59-83. 28647733 10.1159/000469704

[R22] Petazzoni M, Briotti F, Beale B. Unicameral bone cyst of the patella in a young dog. Vet Comp Orthop Traumatol. 2015;28(5):359-63. 26299878 10.3415/VCOT-14-12-0187

[R23] Rosenblatt J, Koder A. Understanding unicameral and aneurysmal bone cysts. Pediatr Rev. 2019 Feb;40(2):51-9. 30709971 10.1542/pir.2015-0128

[R24] Sarierler M, Cullu E, Yurekli Y, Birincioglu S. Bone cement treatment for aneurysmal bone cyst in a dog. J Vet Med Sci. 2004 Sep;66(9):1137-42. 15472481 10.1292/jvms.66.1137

[R25] Thompson KG, Pool RR. Tumors of bones in tumors of domestic animals. USA: Iowa State Press a Blackwell Publishing Company; 2002. p. 245-318.

[R26] Travlos GS. Normal structure, function, and histology of the bone marrow. Toxicol Pathol. 2006;34(5):548-65. 17067943 10.1080/01926230600939856

[R27] Vagias M, Cassidy JP, Skelly C, Mullins RA. Intraosseous epidermoid cysts of adjacent digits in a dog. BMC Vet Res. 2020 Sep 2;16(1):323. 32878616 10.1186/s12917-020-02545-7 PMC7465723

[R28] Vignoli M, Stehlik L, Terragni R, Cavallo L, Proks P. Computed tomography-guided cementoplasty combined with radiation therapy for an aneurysmal bone cyst in a dog: A case report. Vet Med-Czech. 2015 Feb;60(2):109-14.

